# Alveolar ridge height changes in adults and adolescents with angle class I malocclusion before and after orthodontic treatment

**DOI:** 10.4314/ahs.v25i2.30

**Published:** 2025-06

**Authors:** Jun Yin, Jing Li, Yue Li, Yiran Hao, Yuanyuan Li, Yancong Wen, Shixin Cai

**Affiliations:** 1 Department of Stomatology, Hengshui People's Hospital, Hengshui, China; 2 Department of Orthodontics, Hengshui People's Hospital, Hengshui, China; 3 Department of Medicine, Beijing Garrison District Haidian 19th Retired Cadre Rest Center, Beijing, China; 4 Department of Stomatology, The Fifth People's Hospital of Hengshui, Hengshui, China

**Keywords:** Meiotic orthodontic treatment, Alveolar ridge height, Angle Class I malocclusion, Adult, Adolescent

## Abstract

**Background:**

This study compared the changes in alveolar ridge height in adult and adolescent patients with Angle Class I malocclusion before and after orthodontic treatment.

**Methodology:**

A total of 110 patients, 55 adults (18 years and above) and 55 adolescents (10 to less than 18 years), were included. The research focused on alveolar bone thickness and changes in ridge height.

**Results:**

Results showed no significant differences in alveolar ridge thickness between the groups at various locations. Adults had thinner alveolar ridges at 7 mm and 9 mm compared to adolescents. Post-treatment, adults experienced increased ridge height in specific regions, while adolescents had minor changes. Some craniofacial angles and chin concavity differed between the groups. Adults exhibited a higher incidence of bone cracking, with an increase post-treatment. Adolescents didn't show significant changes in bone cracking.

**Conclusion:**

In conclusion, orthodontists should customize treatment for Angle Class I malocclusion based on age-specific findings. Adolescents may experience minor alveolar changes, while adults exhibit increased ridge height and higher fracture risk post-treatment. Monitoring and adapting plans accordingly ensure effective and safe outcomes.

## Introduction

In recent years, the prevalence of Angle Class I malocclusion has exhibited a noticeable increase, characterized by normal relationships between upper and lower dental arches coupled with misaligned teeth. Common manifestations include crowded teeth, protruding front teeth, and interdental gaps^1^,^2^. The structural integrity of the alveolar bone, a pivotal component for dental stability and function, relies on the maintenance of its normal shape. The alveolar ridge's height, defined as the distance from the gingival sulcus to the enamel-cementum junction, is a critical parameter in assessing bone health. A distance exceeding 2 mm between the enamel-cementum junction and the alveolar ridge's summit may signify bone fracture^3^,^4^. Orthodontic interventons play a crucial role in the reconstruction of the alveolar bone and enhancement of soft tissue structures, thereby addressing malocclusions^5^,^6^. Achieving an optimal root-bone relationship is fundamental to obtaining favorable orthodontic outcomes during treatment^7^. While previous studies have explored the impact of orthodontic interventions on Angle Class I malocclusion patients, focusing on changes in alveolar bone quality within the alveolar ridge area, a comprehensive stratified analysis across different age groups has been lacking^8^,^9^. Against the backdrop of human developmental bone dynamics, this study seeks to bridge this gap by investigating variations in alveolar ridge height before and after orthodontic treatment. Specifically, we aim to discern differences between adults and adolescents with Angle Class I malocclusion who have undergone tooth extraction. This research endeavors to shed light on age-specific considerations in the orthodontic management of such cases, contributing valuable insights to the optimization of treatment strategies. Through a meticulous examination of alveolar ridge height changes, we aspire to enhance our understanding of the nuances in orthodontic outcomes across distinct age cohorts, ultimately refining the approach to Angle Class I malocclusion interventions.

## Materials and Methods

### Baseline Information

Fifty-five adult and 55 adolescent patients receiving orthodontic treatment from were enrolled from June 2022 to July 2023. The adult group included patients aged 18 years and older, while the adolescent group included patients aged 10 to 18 years old. There were no statistically significant differences in baseline information between the two groups except for age (P>0.05), as shown in Table 1. This study has been approved by the Ethics Committee of Hengshui People's Hospital (approval number: 2022HSPH-LL-011), and all participants and their families have signed informed consent.

**Table 1:** Baseline Information of enrolled patients [x̅±s, n/(%)]

Group	Age (years)	Sex		Treatment Duration (months)	The severity of Angle Class I Malocclusion	
		
		Male	Female		Mild	Moderate
Adult group (n=55)	23.65±2.15	21(38.18)	34(61.82)	23.16±3.46	33(60.00)	22(40.00)
Adolescent group (n=55)	13.45±2.10	25(45.45)	30(54.55)	22.98±3.15	36(65.45)	19(34.55)
χ^2/t^	25.170	0.598		0.285	0.350	
P	0.000	0.439		0.776	0.554	

### Data Collection

Orthodontic treatment progress was systematically assessed through a standardized data collection protocol. To ensure the accuracy and consistency of measurements, multiple clinicians were involved in the data collection process. Prior to the commencement of the study, all clinicians underwent comprehensive training sessions to standardize their approach and enhance intra-examiner reliability. To minimize potential bias, examiners responsible for outcome assessments were blinded to the treatment status of the patients. Blinding was implemented to prevent preconceived notions or expectations from influencing the evaluation of subjective outcomes, particularly in the case of bone fractures.

#### Inclusion Criteria Exclusion Criteria

The study inclusion criteria were as follows: (1) Patients who have been diagnosed with Angle Class I malocclusion by CT imaging, with a mild to moderate degree of severity10; (2) Patients with normal permanent dentition and periodontal tissues without significant lesions; (3) Patients without pathological root resorption; (4) Patients undergoing orthodontic treatment with self-ligating brackets and extraction of the first premolars. Patients were ineligible if they met the following exclusion criteria: (1) Patients with significant periodontal disease in the active phase; (2) Patients with impacted teeth confirmed by CT imaging; (3) Patients undergoing orthodontic treatment for the second time or more; (4) Patients with missing teeth.

### Treatment Methods

We utilized the Canon Aplio i800 color Doppler ultrasonic diagnostic apparatus with probe models PLI-2004BX (8.8-24.0 MHz) and PLI-1205BX (4-18 MHz). Patients were positioned supine, with metal brackets attached to the clinical crown center points of the upper lateral incisors and first molars on both sides. This procedure was performed by a clinician in our orthodontic department with over 5 years of clinical experience. Patients were instructed to maintain calm respiration, keep their lips closed, and the probe was placed vertically at the line connecting the nostril and the corner of the mouth for longitudinal scanning to display the essence of the incisor and buccal periodontal tissues. Regions of interest (ROI) were outlined, sampling frames were adjusted, and the edges of the ROI were manually delineated. Relevant data were measured, and the average was taken after three repeated measurements.

### Observation Indicators

The following observations were made during the ultrasound examination of the patients: Buccal alveolar bone thickness: The thickness of the buccal alveolar bone was recorded at distances of 5 mm, 7 mm, 9 mm, and 11 mm from the crestal line of the alveolar ridge. Measurements were taken at the following locations: between the first permanent molar and second premolar, near, middle, and far buccal roots of the first permanent molar, and between the first and second permanent molars. Alveolar ridge height: The height of the alveolar ridge was recorded before and after treatment for the central incisors, lateral incisors, and canines in both the upper and lower jaws. Additionally, the condition of the mandible before and after treatment was evaluated, including measurements such as the angle between the sella-nasion-point A (SNA), point A-nasion-point B (ANB), sella-nasion-point B (SNB), angle between the mandibular plane and the occlusal plane, and chin concavity. Bone fractures: The presence of bone fractures was analyzed based on clinical examination. This included observing direct exposure of the alveolar bone and mucosal tissue reaching the top of the alveolar ridge or a distance greater than 2 mm between the cementoenamel junction and the alveolar ridge. These observations were made before and after treatment to assess changes in the dental and skeletal structures of the patients. The thickness of the buccal alveolar bone is measured as the length of the perpendicular line from the surface of the buccal cortical bone to the midpoint of the line connecting the most prominent points on two buccal roots. Alveolar ridge height measurement is the distance from the cementoenamel junction of the tooth on the patient's lip/tongue side to the corresponding alveolar ridge peak on the same side. Chin concavity is the vertical distance between the point of the mandibular foramen and the mandibular border at the most prominent point of the chin.

### Statistical analysis

Data processing was performed using Statistic Package for Social Science (SPSS) 23.0 software (IBM, Armonk, NY, USA). Categorical data such as patient gender, degree of dental malformation, and bone fracture status were expressed as percentages, and the chi-square test was performed. Quantitative data such as alveolar ridge height, mandibular alveolar bone changes, and buccal alveolar bone thickness were expressed as mean ± SDs, and independent sample t-tests were performed between groups, while paired t-tests were used for within-group comparisons. The significance level was set at α=0.05.

## Results

### Comparison of buccal alveolar bone thickness between adults and adolescents

**Figure 1:**
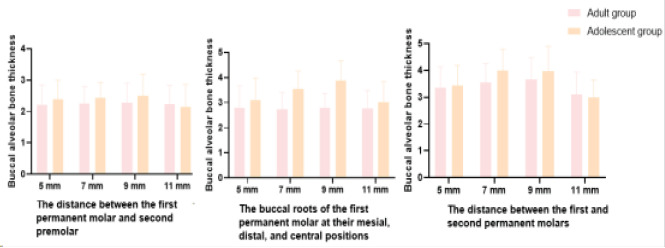
Buccal alveolar bone thickness between adults and adolescents

As shown in Table 2 and Figure 1, we compared the buccal alveolar bone thickness between adults and adolescents at various distances (5 mm, 7 mm, 9 mm, 11 mm) from the first permanent molar to the second premolar. At 5 mm, the buccal alveolar bone thickness in the adult group was 2.21 ± 0.65 mm, while in the adolescent group, it was 2.39 ± 0.61 mm. The t-test result showed a non-significant difference (t = -1.498, p = 0.137). At 7 mm, the thickness in the adult group was 2.26 ± 0.54 mm, compared to 2.44 ± 0.49 mm in the adolescent group. The difference was not statistically significant (t = -1.831, p = 0.070). At 9 mm, the adult group had a thickness of 2.28 ± 0.63 mm, while the adolescent group had 2.51 ± 0.68 mm. The difference was not statistically significant (t = -1.840, p = 0.069). At 11 mm, the thickness in the adult group was 2.24 ± 0.58 mm, and in the adolescent group, it was 2.15 ± 0.71 mm, with no significant difference (t = 0.728, p = 0.468). Also, we compared the buccal roots of the first permanent molar at their mesial, distal, and central positions between adults and adolescents at various distances (5 mm, 7 mm, 9 mm, 11 mm). At 5 mm, the buccal roots thickness in the adult group was 2.81 ± 0.86 mm, while in the adolescent group, it was 3.11 ± 0.87 mm. The difference was not statistically significant (t = -1.819, p = 0.072). At 7 mm, the thickness in the adult group was 2.75 ± 0.65 mm, compared to 3.55 ± 0.72 mm in the adolescent group. The difference was highly significant (t = -6.116, p < 0.001). At 9 mm, the adult group had a thickness of 2.78 ± 0.59 mm, while the adolescent group had 3.87 ± 0.81 mm. The difference was highly significant (t = -8.067, p < 0.001). At 11 mm, the thickness in the adult group was 2.77 ± 0.71 mm, and in the adolescent group, it was 3.01 ± 0.82 mm. The difference was not statistically significant (t = -1.641, p = 0.104). In addition, we compared the distance between the first and second permanent molars between adults and adolescents at various distances (5 mm, 7 mm, 9 mm, 11 mm). At 5 mm, the distance in the adult group was 3.36 ± 0.78 mm, and in the adolescent group, it was 3.45 ± 0.73 mm, with no significant difference (t = -0.625, p = 0.533). At 7 mm, the distance in the adult group was 3.55 ± 0.71 mm, compared to 4.01 ± 0.78 mm in the adolescent group. The difference was highly significant (t = -3.234, p = 0.002). At 9 mm, the adult group had a distance of 3.66 ± 0.82 mm, while the adolescent group had 3.98 ± 0.92 mm. The difference was not statistically significant (t = -1.926, p = 0.057). At 11 mm, the distance in the adult group was 3.11 ± 0.83 mm, and in the adolescent group, it was 2.98 ± 0.67 mm, with no significant difference (t = 0.904, p = 0.368). Overall, these findings provide a detailed examination of buccal alveolar bone characteristics, emphasizing specific distances where significant differences between adult and adolescent groups exist, as reflected by the respective t-values and p-values.

**Table 2:** Comparison of buccal alveolar bone thickness between adults and adolescents (x̅±s, mm)

Group	The distance between the first permanent molar and second premolar			
	5 mm	7 mm	9 mm	11 mm
	
Adult group (n=55)	2.21±0.65	2.26±0.54	2.28**±**0.63	2.24±0.58
Adolescent group (n=55)	2.39±0.61	2.44±0.49	2.51**±**0.68	2.15±0.71
t	-1.498	-1.831	-1.840	0.728
P	0.137	0.070	0.069	0.468

Group	The buccal roots of the first permanent molar at their mesial, distal, and central positions			

	5 mm	7 mm	9 mm	11 mm
	
Adult group (n=55)	2.81±0.86	2.75±0.65	2.78**±**0.59	2.77±0.71
Adolescent group (n=55)	3.11±0.87	3.55±0.72	3.87**±**0.81	3.01±0.82
t	-1.819	-6.116	-8.067	-1.641
P	0.072	0.000	0.000	0.104

Group	The distance between the first and second permanent molars			

	5 mm	7 mm	9 mm	11 mm
	
Adult group (n=55)	3.36±0.78	3.55±0.71	3.66**±**0.82	3.11±0.83
Adolescent group (n=55)	3.45±0.73	4.01±0.78	3.98**±**0.92	2.98±0.67
t	-0.625	-3.234	-1.926	0.904
P	0.533	0.002	0.057	0.368

### Comparison of alveolar ridge height between adults and adolescents before and after treatment

Before and after treatment, there were differences in alveolar ridge height between adults and adolescents in the maxillary and mandibular central incisors, maxillary and mandibular lateral incisors, maxillary and mandibular canines on the labial and palatal sides, and maxillary and mandibular canines on the labial and lingual sides. The alveolar ridge height in all these areas was higher in the adult group than in the adolescent group (P<0.05). After treatment, the alveolar ridge height of the maxillary and mandibular central incisors on the labial and palatal sides, maxillary lateral incisors on the palatal side, mandibular lateral incisors on the lingual side, maxillary canines on the labial side, mandibular canines on the labial and lingual sides in the adult group were higher than before treatment (P<0.05). There was no significant difference in alveolar ridge height before and after treatment in maxillary lateral incisors on the labial side, mandibular lateral incisors on the labial side, and maxillary canines on the palatal side (P>0.05). There was no significant difference in alveolar ridge height in various areas before and after treatment in the adolescent group (P>0.05). Detailed information was shown in Table 3.

**Table 3:** Comparison of alveolar ridge height between adults and adolescents before and after treatment (x̅±s, mm)

Group	Maxillary central incisors labial sides		Maxillary central incisors palatal sides		Mandibular central incisors labial sides	

	Before treatment	After treatment	Before treatment	After treatment	Before treatment	After treatment
Adult group (n=55)	1.38±0.35	1.64±0.32*	1.44±0.33	1.84±0.64*	1.55±0.42	1.88±0.54*
Adolescent group (n=55)	0.81±0.33	0.83±0.36	0.95±0.16	0.93±0.32	0.97±0.33	0.96±0.38
t	8.788	12.472	9.909	9.432	8.053	10.333
P	0.000	0.000	0.000	0.000	0.000	0.000

Group	Mandibular central incisors lingual sides		Maxillary lateral incisors labial sides		Maxillary lateral incisors palatal sides	

	Before treatment	After treatment	Before treatment	After treatment	Before treatment	After treatment

Adult group (n=55)	1.66±0.37	1.96±0.35*	1.71±0.26	1.79±0.24	1.36±0.36	1.88±0.51*
Adolescent group (n=55)	1.02±0.26	1.11±0.33	1.05±0.36	1.06±0.45	0.78±0.28	0.98±0.64
t	10.496	13.104	11.022	10.615	9.431	8.156
P	0.000	0.000	0.000	0.000	0.000	0.000

Group	Mandibular lateral incisors labial sides		Mandibular lateral incisors lingual sides		Maxillary canines labial sides	

	Before treatment	After treatment	Before treatment	After treatment	Before treatment	After treatment

Adult group (n=55)	1.72±0.45	1.88±0.49	1.55±0.41	1.89±0.34*	1.51±0.31	1.96±0.35*
Adolescent group (n=55)	0.87±0.23	0.93±0.45	0.97±0.31	1.05±0.38	1.06±0.45	1.16±0.62
t	12.474	10.590	8.368	12.217	6.107	8.333
P	0.000	0.000	0.000	0.000	0.000	0.000

Group	Maxillary canines palatal sides		Mandibular canines labial sides		Mandibular canines lingual sides	

	Before treatment	After treatment	Before treatment	After treatment	Before treatment	After treatment

Adult group (n=55)	1.55±0.41	1.52±0.43	1.78±0.46	2.11±0.41*	1.55±0.31	1.93±0.29*
Adolescent group (n=55)	0.77±0.31	0.82±0.38	0.98±0.41	1.05±0.36	0.88±0.33	0.98±0.41
t	11.254	9.047	9.628	14.408	10.974	14.029
P	0.000	0.000	0.000	0.000	0.000	0.000

* Compared to the same group before treatment, P < 0.05

### Comparison of mandibular conditions before and after treatment in adult and adolescent patients

Table 4 presents a comparison of mandibular conditions before and after treatment in adult and adolescent patients, focusing on SNB angle, ANB angle, Chin concavity, SNId angle, and MP-FH measurements. In the adult group, SNB angle increased from 75.15±1.78 before treatment to 76.48±1.69 after treatment, while ANB angle decreased from 6.15±0.68 to 5.11±0.98, and Chin concavity increased from 2.99±0.67 to 3.11±0.78. For the adolescent group, SNB angle changed from 75.11±1.16 to 76.44±1.22, ANB angle decreased from 5.45±0.65 to 4.56±0.45, and Chin concavity changed from 3.33±0.89 to 2.66±0.45. The t-test results indicated non-significant changes in SNB and ANB angles for both groups (t = 0.140, p = 0.889; t = 0.142, p = 0.887), but significant improvements in Chin concavity for the adult group (t = -2.263, p = 0.026) and the adolescent group (t = 3.706, p = 0.000). Furthermore, SNId angle for the adult group changed from 77.11±3.45 before treatment to 77.16±3.15 after treatment, and MP-FH changed from 24.22±4.16 to 24.01±5.15. In the adolescent group, SNId angle changed from 76.12±0.68 to 78.98±0.49, and MP-FH changed from 25.66±0.64 to 26.11±0.38. The t-test results indicated a significant increase in SNId angle for the adolescent group (t = 2.088, p = 0.039) and a significant decrease in MP-FH for both the adult group (t = -2.537, p = 0.013) and the adolescent group (t = -4.280, p = 0.000). Notably, the asterisks denote significant differences compared to the same group before treatment (P < 0.05). The observed changes in mandibular conditions, including alterations in SNB and ANB angles, as well as variations in chin concavity, hold significant clinical implications for both facial aesthetics and orthodontic treatment outcomes. An increase in SNB angle may suggest mandibular protrusion, impacting facial profile aesthetics, while a decrease in ANB angle may influence bite alignment and potentially lead to malocclusions. Changes in chin concavity can affect chin projection and overall facial harmony. Additionally, variations in SNId angle and MP-FH measurements indicate shifts in vertical facial proportions, which have implications for overall facial height and harmony. These changes underscore the need for tailored orthodontic interventions to optimize facial aesthetics, occlusion, and overall oral health. Understanding these implications ensures that treatment plans address not only functional improvements but also contribute to enhanced facial aesthetics and long-term patient satisfaction.

**Table 4:** Comparison of mandibular conditions before and after treatment in adult and adolescent patients (x̅±s)

Group	SNB angle		ANB angle		Chin concavity	

	Before treatment	After treatment	Before treatment	After treatment	Before treatment	After treatment
Adult group (n=55)	75.15±1.78	76.48±1.69	6.15±0.68	5.11±0.98	2.99±0.67	3.11±0.78
Adolescent group (n=55)	75.11±1.16	76.44±1.22*	5.45±0.65	4.56±0.45*	3.33±0.89	2.66±0.45*
t	0.140	0.142	3.469	3.782	-2.263	3.706
P	0.889	0.887	0.001	0.000	0.026	0.000



Group	SNId angle		MP-FH	

	Before treatment	After treatment	Before treatment	After treatment
Adult group (n=55)	77.11±3.45	7**7**.16±3.15	24.22±4.16	24.01±5.15
Adolescent group (n=55)	76.12±0.68	78.98±0.49*	25.66±0.64	26.11±0.38
t	2.088	-4.234	-2.537	-4.280
P	0.039	0.000	0.013	0.000

* Compared to the same group before treatment, P < 0.05

### Bone fracture conditions before and after treatment

Table 5 presents the bone fracture conditions before and after treatment, reported as frequencies and percentages for the adult and adolescent groups. In the adult group, the incidence of bone fractures increased significantly from 25.45% (14 cases) before treatment to 60.00% (33 cases) after treatment, as evidenced by a chi-square test (χ^2^ = 13.411, p = 0.000). Conversely, in the adolescent group, the incidence of bone fractures saw a slight non-significant rise from 1.82% (1 case) before treatment to 5.45% (3 cases) after treatment (χ^2^ = 1.038, p = 0.308). The overall chi-square test (χ^2^) comparing bone fracture conditions before and after treatment across both groups was 13.046 (p < 0.001), indicating a significant association between treatment and the occurrence of bone fractures. These findings highlight distinct patterns in bone fracture outcomes between adult and adolescent patients, supported by the corresponding chi-square values and p-values.

**Table 5:** Bone fracture conditions before and after treatment (n%)

Group	Before treatment	After treatment	χ^2^	P
Adult group (n=55)	14(25.45)	33(60.00)	13.411	0.000
			
Adolescent group (n=55)	1(1.82)	3(5.45)	1.038	0.308
			
χ^2^	13.046	37.162		
P	0.000	0.000		

## Discussion

The number of individuals with Angle Class I malocclusion undergoing orthodontic treatment is increasing. In the past, clinical evaluations of treatment outcomes were primarily based on differences in alveolar ridge height and alveolar bone thickness. However, there has been limited focus on differences in treatment outcomes among patients in different age groups. In order to achieve personalized treatment goals, this study analyzed the changes in alveolar ridge height before and after orthodontic treatment in adult and adolescent patients with Angle Class I malocclusion.

This study conducted measurements based on the distribution of buccal bone mass in the dental arch. Due to the gradual increase in biting force from anterior to posterior, the functional thickness of the bone increases. The buccal alveolar bone thickness shows a trend of increasing from mesial to distal in three regions, with the greatest thickness observed between the first and second permanent molars^11^,^12^. This study also found that in the buccal root region of the first permanent molar at the mesial and distal aspects, the adult group had smaller buccal alveolar bone thickness at 7 mm and 9 mm compared to the adolescent group. Between the first and second permanent molars, the adult group had smaller buccal alveolar bone thickness at 7 mm compared to the adolescent group. This indicates that bone thickness gradually decreases with age, and the buccal alveolar bone thickness is greater in adolescents than in adults. Considering individual differences in the anatomical position of the maxillary sinus and combining the results of this study, it is necessary to maintain a relatively stable distance between the enamel-bone interface and the alveolar ridge crest before and after tooth extraction orthodontic treatment, and to initiate orthodontic treatment as early as possible. It should be noted that when the alveolar bone is too thin, it can cause false positive results in imaging due to limitations in voxel resolution^13^.

This study found that orthodontic treatment can significantly improve the alveolar ridge height in adult patients, while the changes in alveolar ridge height in adolescent patients before and after treatment are relatively small. The height of the alveolar bone increases with tooth eruption, accompanied by corresponding absorption and apposition on the surface of the mandible, resulting in an overall increase in mandibular bone volume^14^,^15^. The mandibular alveolar bone grows with age and undergoes remodeling. The results of this study suggest a greater risk of bone defects in the anterior tooth region of adults, highlighting the need for early orthodontic treatment during adolescence. Additionally, during the orthodontic process, it is important to correctly apply the principles of mechanical systems to avoid alveolar bone loss in specific areas and improve treatment outcomes^16^.

This study found that orthodontic treatment can have a certain impact on the craniofacial bone structure, and adult patients have a higher risk of bone cracking. Therefore, personalized treatment based on the patient's age and bone structure characteristics is needed for safe and effective orthodontic treatment. During the peak growth period of the mandible in adolescence, the amount of growth significantly decreases after this period^17^. Adolescent patients are at a good stage of alveolar bone development, and their craniofacial bones are in a period of rapid growth and remodeling^18^. Orthodontic treatment during adolescence can reduce the difficulty of correction and improve jaw relationships. Based on the results of this study, timely correction of upper central incisors with lingual inclination in Angle Class I malocclusion patients during adolescence can effectively release the growth potential of the mandible and guide the coordinated growth of the mandible and mandibular alveolar bone. This can also promote mandibular growth and remodeling, leading to mandibular reshaping. Bone cracking is a common complication of Angle Class I malocclusion, and the cases included in this study showed clinical manifestations such as dental crowding and local bone loss caused by crowding, which can increase the risk of bone cracking^19^,^20^. According to the development rules of bone and skeleton, bone growth and density increase significantly from birth to 30 years old. In terms of implant site selection, the best site for adults is between the buccal roots of the first maxillary molar, while for adolescent patients, it is between the first and second maxillary premolars.

Limitations of this study should be acknowledged. Firstly, the sample size, although balanced between adult and adolescent groups, may not fully represent the heterogeneity within each age category. Additionally, the age range for adolescents is relatively broad (10 to less than 18 years), potentially overlooking developmental variations within this group. The focus on Angle Class I malocclusion may limit the generalizability of findings to other malocclusion types. Furthermore, the study primarily assessed alveolar ridge height and bone thickness without considering other factors that could influence treatment outcomes, such as genetics or systemic health. The retrospective nature of the study design introduces the possibility of uncontrolled confounding variables. Lastly, the observed differences in bone cracking incidence may necessitate further investigation into contributing factors, such as treatment techniques or patient compliance. Despite these limitations, the study provides valuable insights into age-specific variations in orthodontic treatment outcomes for Angle Class I malocclusion. Future research avenues could explore long-term stability, genetic and systemic influences, comparative studies across malocclusion types, the impact of treatment techniques, patient-reported outcomes, and the mechanisms underlying bone cracking differences. These directions aim to enhance our understanding and guide more personalized and effective orthodontic care.

In summary, orthodontic treatment can significantly improve the alveolar ridge height in adult patients and have a certain impact on the craniofacial bone structure. However, adolescent patients have relatively small changes in alveolar ridge height. In addition, adult patients have a higher risk of bone cracking. Therefore, individualized treatment planning based on the patient's age and bone structure characteristics is necessary to ensure the effectiveness and safety of orthodontic treatment.

## Figures and Tables

**Figure 2:**
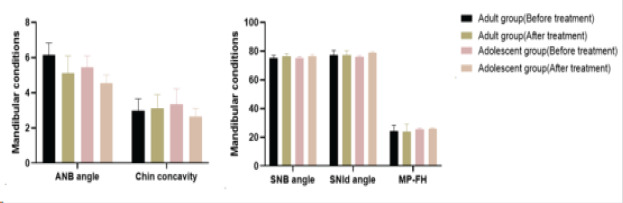
Mandibular conditions before and after treatment in adult and adolescent patients
